# How to design a registry for undiagnosed patients in the framework of rare disease diagnosis: suggestions on software, data set and coding system

**DOI:** 10.1186/s13023-021-01831-3

**Published:** 2021-05-01

**Authors:** Alexandra Berger, Anne-Kathrin Rustemeier, Jens Göbel, Dennis Kadioglu, Vanessa Britz, Katharina Schubert, Klaus Mohnike, Holger Storf, Thomas O. F. Wagner

**Affiliations:** 1 Frankfurt Reference Centre for Rare Diseases, University Hospital Frankfurt, Goethe University Frankfurt, Theodor-Stern-Kai 7, 60590 Frankfurt am Main, Germany; 2 Medical Clinic II, University Hospital Gießen and Marburg, Klinikstraße 33, 35392 Gießen, Germany; 3 Medical Informatics Group Frankfurt, University Hospital Frankfurt, Goethe University Frankfurt, Theodor-Stern-Kai 7, 60590 Frankfurt am Main, Germany; 4 Central-German Network for rare diseases, University Hospital Magdeburg A.Ö.R, Leipziger Straße 44, 39120 Magdeburg, Germany

**Keywords:** Registry, Undiagnosed patients, Rare diseases, HPO

## Abstract

**Background:**

About 30 million people in the EU and USA, respectively, suffer from a rare disease. Driven by European legislative requirements, national strategies for the improvement of care in rare diseases are being developed. To improve timely and correct diagnosis for patients with rare diseases, the development of a registry for undiagnosed patients was recommended by the German National Action Plan. In this paper we focus on the question on how such a registry for undiagnosed patients can be built and which information it should contain.

**Results:**

To develop a registry for undiagnosed patients, a software for data acquisition and storage, an appropriate data set and an applicable terminology/classification system for the data collected are needed. We have used the open-source software Open-Source Registry System for Rare Diseases (OSSE) to build the registry for undiagnosed patients. Our data set is based on the minimal data set for rare disease patient registries recommended by the European Rare Disease Registries Platform. We extended this Common Data Set to also include symptoms, clinical findings and other diagnoses. In order to ensure findability, comparability and statistical analysis, symptoms, clinical findings and diagnoses have to be encoded. We evaluated three medical ontologies (SNOMED CT, HPO and LOINC) for their usefulness. With exact matches of 98% of tested medical terms, a mean number of five deposited synonyms, SNOMED CT seemed to fit our needs best. HPO and LOINC provided 73% and 31% of exacts matches of clinical terms respectively. Allowing more generic codes for a defined symptom, with SNOMED CT 99%, with HPO 89% and with LOINC 39% of terms could be encoded.

**Conclusions:**

With the use of the OSSE software and a data set, which, in addition to the Common Data Set, focuses on symptoms and clinical findings, a functioning and meaningful registry for undiagnosed patients can be implemented. The next step is the implementation of the registry in centres for rare diseases. With the help of medical informatics and big data analysis, case similarity analyses could be realized and aid as a decision-support tool enabling diagnosis of some undiagnosed patients.

## Background

A rare disease is a health condition that affects a small number of people compared with other prevalent diseases in the general population. While there is no universal definition of rare diseases, the concept of rare diseases in the current political and legislative framework is closely linked to a definition by point prevalence. Most jurisdictions include a prevalence threshold in at least one definition of rare diseases, whereas incidence as specification in a rare disease definition is seldomly used [1].

In the European Union (EU), a disease is considered to be rare, if it affects less than 5 of 10,000 people. In the United States of America (USA) a rare disease is defined as affecting less than 200,000 inhabitants, translating to a prevalence of about 8–9 out of 10,000 people [2]. About 30 million people in both the EU and the USA are suffering from a disease that is considered a rare disease [3, 4].

Orphanet is a 37-country network, aiming to increase knowledge of rare diseases. It was cofounded by the European Commission in 1997. As of 2020, classification and descriptions of 6172 rare diseases (by the European definition) are included in the Orphanet database; 71.9% being genetic and the onset of symptoms occurring during childhood in 69,9%. About 85% of rare diseases are ultra-rare with a prevalence of less than 1 per 1,000,000 [2, 5].

Due to insufficient epidemiological data, lack of scientific publications and an absence of structured databases, the number of patients suffering from an uncharacterized disease is hard to estimate. In terms of uncharacterized genetic diseases, estimates state an unidentified underlying disease gene for at least 3,000 human Mendelian diseases, and the true number may be much higher [6].

Many rare diseases are severe chronic conditions with a complex clinical presentation and a negative impact on life expectancy and quality of life [7]. Prevention and cure as well as adequate therapies exist only for a minority of rare diseases [8]. Therefore, patients with rare diseases face a multitude of disease-related problems. Starting with delayed diagnosis, multiple doctor’s visits before a diagnosis is made, misleading diagnosis, lack of comprehensive information provided at the time of diagnosis, insufficient coordination of care, inadequate transition from paediatric to adult care, and low or non-existent access to medication due to poor knowledge or lacking research and clinical trials. Patient organizations play a vital role in improving these circumstances [9, 10].

The diagnostic odyssey, that many patients affected by rare diseases experience, is often due to multiple causes: a non-specific clinical presentation involving multiple organ systems that seem to be unrelated, a general lack of awareness and physician training regarding rare diseases, missing standard diagnostic criteria, a limited number of specialists, uncoordinated patient journeys through the health-care system, that cause loss of information and increase the possibility of errors and sometimes limited access to diagnostic tools [11–13].

### The connection between undiagnosed and rare disease patients

It is important to state, that rare diseases remain not always undiagnosed and undiagnosed diseases are not always hidden rare diseases. The undiagnosed patient can be affected by a rare disease, a more common disease that presents atypically, by multiple diseases occurring simultaneously, including psychosomatic disorders or by a completely new and uncharacterized disease. Both undiagnosed and rare disease patients require broad interdisciplinary evaluation, access to modern information resources and special diagnostic techniques including molecular genetics [14]. Therefore, the centres for rare diseases across Germany offer visiting hours for undiagnosed patients with or without a suspected rare disease.

In terms of the diagnostic process, a  diagnosis can be delayed when the patient has not yet been referred to the appropriate expert. This can be caused by gatekeeping delays in primary care due to missing knowledge about rare diseases as well as systemic problems due to a lack of coordination, collaboration and adequate exchange of information between several healthcare providers [11, 15–17]. A complex diagnosis is defined by a non-conclusive phenotype and genomic profile, insufficient biomarkers, presentation of unspecific but common symptoms or the concurrent existence of more than one disease. In this case, the patient might require specific equipment and contact with a centre of expertise or a reference network. In case of a diagnostic impasse all available investigations have been carried out by experts and the patient and physicians may be facing a new, yet undescribed disorder [3, 11, 16, 17].

### Actions for rare diseases

Initiated by patient organizations, rare diseases have gained attention in politics over the last decade. Driven by European legislative requirements, national strategies for the improvement of care in rare diseases had to be developed [18]. In Germany, the National Action Plan for People with Rare Diseases implemented 52 measures to improve health care for patients with rare diseases. Some examples are: Recommendations for the implementation of national centres of expertise, specific measures to accelerate time to diagnosis, research support, improvement of information management as well as suggestions on financing of these measures [19].

Concerning research, the development of a registry toolbox for creating individual disease-specific registries was requested. This registry toolbox should make use of an open-source software with a defined minimal data scheme and an emphasis on interoperability on a national and international level as well as metadata management [19]. This project was conducted collaboratively by the Institute of Medical Biometrics, Epidemiology and Informatics of the University Medical Centre of the Johannes Gutenberg University Mainz and the University Hospital Frankfurt in 2013 as part of the German National Action Plan and yielded the “Open Source Registry System for Seltene Erkrankungen (OSSE)”. OSSE is an easily scalable and customizable framework for developing disease specific rare disease registries automatically connected to a meta data repository and fulfilling the FAIR data principles [20]: Findable: By describing metadata, people and computers can interact with the data to search for specific records. Accessible: Data is stored long-term, with defined license and access conditions, both at the level of metadata as well as the level of the instance data. Interoperable: Data sets can be combined with other data sets. Reusable: Data can be used for further research using computational methods. Further development of the OSSE registry framework is ongoing by the Medical Informatics Group (MIG) of the University Hospital Frankfurt [19, 21–23].

To improve timely and correct diagnosis for patients with rare diseases, the development of a ‘registry for undiagnosed patients’ was also recommended by the German National Action Plan, taking into account that a high percentage of these ‘undiagnosed patients’ eventually are diagnosed to have a rare disease [19].

Similar National strategies have been developed in most member states of the European Union as well as Norway, Switzerland and the UK [24] Some international examples are: The National Institutes of Health Undiagnosed Diseases Program, which started in 2008 [25]; the “Nan-Byo” (which translates as “difficult and illness”), which was established in 1972 in Japan and extended in 2015 as Japan’s Initiative on Rare and Undiagnosed Diseases [26]; In February 2020, the Australian government announced to provide funding for activities to implement the National Strategic Action Plan for Rare Diseases, which was developed by Rare Voices Australia [27].

### Registries for rare diseases

Registries in general and especially in the field of rare diseases can help to connect data from multiple health care providers (HCP), thus enlarging the data base for research questions, including epidemiology of rare diseases. However, disease-specific ICD-10 codes are not available for most rare diseases and Orpha-codes, OMIM-codes or alpha-IDs are not used in routine clinical care. Therefore, prevalence calculated from disease-specific registries have limited accuracy [28, 29]. And, of course, usually academia driven registries do not achieve sufficient representation of the whole disease population to allow calculation of prevalence.

Due to the fact that undiagnosed patients present with a wide variety of symptoms at different levels and specialities within the health care system, it is even more complicated to assess the number of undiagnosed patients.

Undiagnosed patients face specific problems caused by their lack of diagnosis. Such as long diagnostic odysseys and also a feeling of “not belonging anywhere” and self-doubt, which prevents access to self-help-groups and social support. Illustrating the feelings of suffering and loss, the inability to make plans, uncertainty, fear and rejection by clinicians and others, illness narratives of undiagnosed patients are from a chaotic type [30]. Therefore, it seems reasonable to create a registry addressing undiagnosed patients in order to create an opportunity to connect with others experiencing similar problems, shorten their path to diagnosis and by identifying chronic conditions at an earlier stage possibly producing savings to the health care system [31].

As most medical registries focus on one specific disease or group of diseases, they contain disease-specific and disease-relevant data. Patients, who are not yet diagnosed do not fit into these registry schemes. Therefore, in this paper we focus on the question on how such a registry for undiagnosed patients can be built and which information it should contain.

## Methods

According to joint recommendations on how to improve the quality of rare disease registries, the first step is to classify the registry and to define its purpose and key stakeholders [32].

### Classification of the registry and definition of objectives

The registry for undiagnosed patients is primarily a clinical registry focusing on the natural course of a group of diseases, namely ones, that are seemingly hard to recognize. It can be used to estimate the number of rare disease patients among the group of undiagnosed patients and, if operated nationwide, can aid to estimate the prevalence of undiagnosed patients, thus serving public health issues. It is a non-population-based registry, based on clinical centres for patients with rare (and undiagnosed) diseases. Inclusion criteria are: all patients presenting to a rare disease centre in search for a diagnosis who have given informed consent to participate in the registry, regardless of whether a rare disease is suspected or not. Exclusion criteria is a confirmed diagnosis of one or several diseases that explain all symptoms. The registry for undiagnosed patients is a mainly physician-driven registry, in which data is entered manually.

The primary objective of our proposed registry for undiagnosed patients is to describe the population of patients, that remain undiagnosed and accompany them on the path to diagnosis while describing the natural course of their disease.

Secondary objectives are to facilitate research regarding rare diseases: As soon as a patient is diagnosed with a rare disease and agrees to data-sharing, the collected data set can be transferred to a disease-specific registry, if such a registry exists. This helps in gaining patients and data for disease-specific research questions as well as connecting different centres of expertise to work together more closely. Another objective is to help diagnosing patients earlier based on case-similarity analysis. By comparing the current and past clinical symptoms, objective findings and diagnoses of new patients with those who already received a diagnosis, possible similarities could mean, that the underlying diseases are the same. Additionally, very similar case histories of a number of undiagnosed patients could also help to identify patient cohorts for further targeted research.

Possible future objectives may also be the description of patient journeys and identifying structural problems in the health-care-system and to assess the quality of care of the particular centres of rare diseases, for example by including patient satisfaction reports as well as connecting undiagnosed patients and empowering them to advocate their needs in society. For these purposes though, new modules of data sets need to be developed.

With these objectives in mind, the key stakeholders of our proposed registry are the patients, the physicians treating them and the researchers in the centres for rare diseases. Despite the recommendation of Kodra et al. [32] to include all key stakeholders from the beginning in the process of developing a registry, we did not include patients in this process, as currently no patient-organization for undiagnosed patients exists in Germany.

To develop a registry for undiagnosed patients, a software for data acquisition and storage, an appropriate data set and an applicable terminology/classification system for the data used is needed [32].

### Registry software

We have used the open-source software OSSE [21] as a framework for the registry for undiagnosed patients. This software enables users—even with limited IT-knowledge—to create registry data schemes for the individual purpose. The data items are specified as data elements in a metadata storage, where they can be retrieved to be re-used as templates in future registries. This openly accessible metadata, and the possibility for researchers to get an impression of the data a registry collects, without forcing the registry to centrally disclose their data allow for a wide interoperability with other registries and research facilities. This in turn enables the researcher on the one hand to decide, which registries can provide appropriate data and on the other hand formulate a detailed inquiry for data using a so called OSSE decentral search inquiry [23].

Another strong point of the OSSE software is data protection. For pseudonymization, OSSE uses a broadly established open-source software, Mainzelliste, developed by the University Medical Centre of the Johannes Gutenberg University Mainz [33, 34]. OSSE also offers templates for patient information and declaration of consent. For further information on the software see https://www.osse-register.de/en/ [21].

As mentioned above, OSSE complies with the FAIR data principles, ensures data protection, is easy to use by registry personnel, was developed by IT-specialists on our site and therefore fulfils all requirements of an IT-system according to the recommendations of Kodra et al. [32].

### Data set and coding

The basis of our work was the minimal data set for rare disease patient registries recommended for European cooperation Version 3.0 (see. Table 1). Version 3.0 differs only in minor aspects from Version 0.1 [35], which itself has been built based on the French minimal data set *RD MDS v1.08* [36].

**Table 1:** Data set for rare disease patient registries recommended for European Cooperation (Version 3.0), based on the French minimal data set RD MDS v1.08

Item group	Item no	Item concept	Question	Content coding	Data collection (one-time/repeatedly)	Comment
1. Pseudonym	1.1	Patient’s Pseudonym (PID)	Patient’s Pseudonym (as defined in the meta-data-set)	String	One-time	
2. Personal Information	2.4	Patient’s date of birth	Patient’s date of birth as recorded on the birth certificate	Date	One-time	
	2.5	Gender	Patient’s gender	Female Male Undetermined Unknown (for the foetus)	One-time	
3. Family Information	3.1	Patient born from a relationship between related parties	Is the patient born from a relationship between related parties	Yes No Unknown	One-time	
4. Vital status	4.1	Patient’s vital status	Is the patient still alive?	Yes No Lost to follow-up Discharged from registry	Repeatedly	Update of the data base at least once per year
	4.2	Patient’s date of death	Patient’s date of death	Date	One-time	Update of the data base at least once per year
	4.3	Death due to rare disease	Is the death due to the rare disease the patient is suffering from?	Yes No Unknown		
5. Care pathway	5.1	Patient’s date of inclusion in the registry	Date at which the patient was included in the registry	Date		
6. Disease history	6.1	Age at onset	Age at which symptoms first appeared	Antenatal At birth XX year (s) and XX month (s) Undetermined		
	6.2	Age at diagnosis	Age at which the diagnosis was made	Antenatal At birth XX year (s) and XX month (s) Undetermined		
7. Diagnosis	7.2	Diagnosis of the rare disease	Diagnosis retained by the RD center	Alpha code		
8. Research	8.1	Agreement to be contacted for a protocol	Does the patient give permission to be contacted for a research protocol?	Yes No		
	8.2	Patient non-opposition to the reuse of data	Does the patient give permission for his/her data to be reused for other research purposes?	Yes No		
	8.3	Patient having previously given a biological sample for research	Has the patient already given a biological sample for research?	Yes No		
	8.4	Patient having previously given a biological sample for molecular diagnosis	Has the patient already given a biological sample for molecular diagnosis?	Yes No		

Two clinicians/researchers and two study nurses decided, how to expand this data set and which terminologies to use. They were advised by IT-personnel regarding further ideas and practicability in a course of repeated meetings over months. As mentioned above, no patients or patient representatives were included in this process.

As rare diseases are heterogenous and complex in their clinical presentation, we decided to extend the minimal data set by (subjective) symptoms and (objective) clinical findings together with the time of their first presentation as well as established or suspected diagnoses in each patient. This information can easily be obtained from prior medical records and the patient’s history, which all patients have to provide when addressing rare disease centres to have their diagnostic halt overcome. Matching the information in prior medical files to the information, the patient gives directly (via interview or checklists) helps to validate the information and check for reliability. In order to ensure comparability and statistical evaluation, symptoms, clinical findings and diagnoses have to be encoded. Therefore, the registry forms have to be filled out at least partially by medical staff.

As the ICD-10 code does not have sufficient specificity and granularity for rare diseases, we included the Alpha ID [37] and Orpha Code [38] to encode rare diseases in the registry for undiagnosed patients.

We evaluated three terminologies resp. ontologies (in the following coding systems), i.e. Systematized Nomenclature of Medicine—Clinical Terms (SNOMED CT) [39], Human Phenotype Ontology (HPO) [40] and Logical Observation Identifiers Names and Codes (LOINC) [41] with regard to usefulness and feasibility for a registry for undiagnosed patients.

The Frankfurt Reference Centre for Rare Diseases (FRZSE), among other activities, runs a students’ clinic for patients without a diagnosis, where patient cases are discussed in interdisciplinary teams to eventually find a diagnosis.

We used 10 random patient files from this students’ clinic to evaluate the developed data set and to compare the 3 different coding systems. All identifying data of the files were removed, thus ensuring data protection by anonymization.

All symptoms, diagnoses and clinical findings mentioned in the medical records were extracted by one researcher and translated into English. Each symptom was extracted only once, regardless of how often it was mentioned in the file. Overall, 80 medical terms were extracted. These terms were entered in the browsers of each of the 3 coding systems. In some cases, more than one possible translation was entered into the browser to increase the chance of a match.

We evaluated the classification systems by numbers/percentages of matches for the extracted medical terms. A term could be an exact match, a more general match, a match that is too specific, a match for which a quantitative figure is needed, or no match. This decision was made by the same researcher, who extracted the medical terms from the files.

## Results

### Evaluation of coding systems

With SNOMED CT, 98% of the 80 medical terms could be coded correctly. For one term, *increase in waist circumference*, an exact quantitative information is mandatory. Elevated gamma-glutamyl transferase levels could not be coded with SNOMED CT. For every medical term that could be coded correctly a mean number of 5 synonyms (range 2–15) was provided by SNOMED CT.

HPO provided exact matches for 73% of the terms. More general codes are available for 16% and codes that were too specific for 3% of the terms. 9% of the medical terms could not be coded. Only about 2 synonyms for each term are available so that further synonyms had to be entered manually to increase finding matches.

Only 31% of medical terms were coded correctly by the LOINC nomenclature. Generic codes are available for 8%, too specific ones for 33% of the terms. 8% of the medical terms could only be coded with an exact quantitative measurement. 21% of medical terms could not be coded at all. Therefore, symptoms and clinical findings cannot be coded sufficiently with LOINC. Lab values can only be represented with their exact value. Basic changes in lab values, for example hyponatraemia, cannot be described with LOINC. Medical terms and their synonyms had to be entered manually in most instances because LOINC only provides a mean of one synonym per term.

**Fig. 1:**
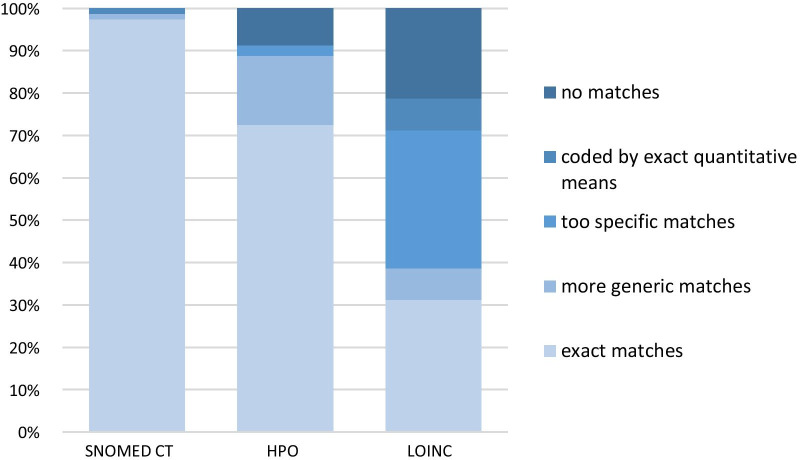
Evaluation of classification systems regarding matches for extracted medical terms

Under the assumption that also more generic coding terms are acceptable, 99% of medical terms are matched with SNOMED CT, 89% with HPO and 39% with LOINC (see Fig. 1).

### Data set

The development of the data set for the undiagnosed patients’ registry was based on the minimal data set for rare disease patient registries recommended for European cooperation Version 3.0. [35] (see Table 1) This data set had been developed for the EUCERD-Action of the European Commission and has been the basis for the Set of Common Data Elements for Rare Diseases Registration, which was released by the EU Rare Disease platform with only some minor changes [42].

We extended the minimal data set by symptoms, clinical findings and prior diagnoses and generated two forms: a basic form, which is filled out initially when a patient is included in the registry and an episodic or longitudinal form, which can be filled out several times. (see Table 2). The basic form can only be altered by an administrator once the data set has been saved. The episodic form functions as a follow-up tool. The forms have to be filled out by personnel of the rare disease centre, for example study nurses or clinicians/researchers and may be included into the patients’ medical files.

**Table 2:** Suggested data set for a registry for undiagnosed patients

Item group	Item No	Item concept	Concept description	Content coding
1. Personal data/information	1.1	Patient's first name	Patient's first name as specified on the birth certificate or identity card	
	1.2	Patient's (married) last name	Patient's (married) last name	
	1.3	Patient's birth name	Patient's birth name	
	1.4	Patient's date of birth	Patient's date of birth as recorded on the birth certificate and whether Information is recorded for a foetus	
	1.5	City of residence	Patient's current city of residence	
	1.6	Gender	Patient's gender	Female Male Undetermined Unknown (for the foetus)
2. Family information	2.1	Patient born from a relationship between related parties	Is the patient born from a relationship between related parties?	Unknown No, suspected No, confirmed Yes, suspected Yes, confirmed
3. Vital Status	3.1	Patient's vital status upon inclusion into the registry	Is the patient still alive?	Alive Dead
	3.2	Patient's date of death	Patient's date of death	
4. Care Pathway	4.1	Patient's date of inclusion in the RD centre	Date at which the patient was recorded in the RD centre. Please enter the date when the patient was included in the internal medical information system	
5. Disease history	5.1	Point in time at onset	When were the symptoms first noticed? (Only fill date input fields if "lifetime" was selected)	Antenatal At birth Undetermined lifetime '*Year of first manifestation'* *'Month of first manifestation'* *'Day of first manifestation*
6. Diagnosis upon inclusion into the registry	6.1	Diagnosis code	Prior Diagnosis (Code)	
	6.2	Type of code	Specify which type of code is used—use ICD-10 if possible-	ICD-10 Alpha-ID Orphacode
	6.3	Description of the chosen Code	Description of the chosen code. Please copy the EXACT text belonging to the code. Do NOT enter free text	
	6.4	Status of diagnosis	Specify whether the diagnosis is already confirmed or only suspected	Unknown Confirmed Suspected
7. Symptom history	7.1	Diagnosis code (Symptom)	Diagnosis (code) of the symptom	
	7.2	Type of code (Symptom)	Specify which type of code is used—use HPO if possible-	HPO SNOMED-CT
	7.3	Symptom Ontology description	Description from the selected code	
	7.4	Symptom priority	Symptom priority	Unknown High Medium Low
	7.5	Year of first manifestation	Year of first manifestation	
	7.6	Month of first manifestation	Month of first manifestation	Unknown January February March April May June July August September October November December
E1. Vital Status	E1.1	Patient's vital status	Is the patient still alive	Alive Dead Lost to follow up Discharged from registry
	E1.2	Patient's date of death	Patient's date of death	
	E1.3	Death due to the rare disease	Is the death due to the rare disease the patient is suffering from?	Yes No Unknown
E2. Further Symptoms		Diagnosis code (Symptom)	Diagnosis (code) of the symptom	
	E2.1	Type of code (Symptom)	Specify which type of code is used—use HPO if possible-	HPO SNOMED-CT
	E2.2	Symptom Ontology description	Description from the selected code	
	E2.3	Symptom priority	Symptom priority	Unknown High Medium Low
	E2.4	Year of first manifestation	Year of first manifestation	
	E2.5	Month of first manifestation	Month of first manifestation	Unknown January, February March, April May, June July, August September October November December
E3. Further Diagnosis	E3.1	Diagnosis code	Prior Diagnosis (Code)	
	E3.2	Type of code	Specify which type of code is used—use ICD-10 if possible-	ICD-10 Alpha-ID Orphacode
	E3.3	Description of the chosen Code	Description of the chosen code. Please copy the EXACT text belonging to the code. Do NOT enter free text	
	E3.4	Status of diagnosis	Specify whether the diagnosis is already confirmed or only suspected	Unknown Confirmed Suspected
E4. Causal Diagnosis (final)	E4.1	Diagnosis code	Diagnosis (Code)	
	E4.2	Type of code	Specify which type of code is used—use ICD-10 if possible-	ICD-10 Alpha-ID Orphacode
	E4.3	Description of the chosen Code	Description of the chosen code. Please copy the EXACT text belonging to the code. Do NOT enter free text	
	E4.4	Status of diagnosis	Specify whether the diagnosis is already confirmed or only suspected	Unknown Confirmed Suspected
	E4.5	Rare Disease	Is the newly found diagnosis a rare disease?	(Click box)
	E4.6	Year of diagnosis	Year of diagnosis	
	E4.7	Month of diagnosis	Month of diagnosis	Unknown January February March April May June July August September October November December
E5. Research	E5.1	Agreement to be contacted for a protocol	Does the patient give permission to be contacted for a research protocol?	Yes No Unknown
	E5.2	Patient non-opposition to the reuse of data	Is the patient non-opposite to the reuse of data?	Yes No Unknown
	E5.3	Patient having previously given a biological sample for research	Has the patient already given a biological sample for research?	Yes No Unknown

### The basic form

Personal information such as name, surname, date of birth and current address, more precisely the postal code, is used to create the personal ID (pseudonym) with the Mainzelliste.

Gender of the patient is documented.

As over 70% of rare diseases have a genetic origin, we ask for the patient´s parents’ consanguinity in the family information.

We ask for vital status upon inclusion in the registry. If the patient has already died, the date of death is to be entered, too. Date of inclusion into the registry is reported.

For the disease history, we ask for the time when the first symptoms were noticed.

Previously diagnosed diseases are prompted. These diagnoses shall be coded with an international disease classification, preferably with the ICD-10. When a more specific code is needed, for example when a coexisting rare disease is to be coded, Alpha-ID or Orpha Code, can be used. Therefore, the type of code as well as its description is to be entered as well. A statement whether the diagnosis has been confirmed or is still being suspected is asked for.

Symptoms and clinical findings are to be entered together with the type of nomenclature with which they are coded and an exact description of the term in the coding system. Year and month of the symptoms’ first appearance are to be entered as well as its impact on the patient`s life.

### The episodic form

Together with the basic form, the first longitudinal form has to be completed. When an episodic form is created, the data collection date has to be entered. Then vital status, date of death and if death was caused by a rare disease, if applicable, are to be filled out.

Newly developed symptoms and further diagnoses can be entered as well as those that were missed to be documented during the first data collection. The number of symptoms and diagnoses that can be entered is not limited.

If a causal diagnosis is found which potentially explains all symptoms, this information can be recorded as well. The diagnosis itself shall again be coded preferably using the ICD-10. Type and description of the code, status of diagnosis, year and month of diagnosis and whether the disease is a rare one shall be entered.

Lastly, information for research questions is to be entered. As these can change over time, only the latest episodic form is applicable. The questions concerning research are:

1. Has the patient given consent to be contacted for a study protocol?

2. Has the patient been informed concerning and not opposed to a future re-use of his/her de-identified data for other research purposes?

3. Has the patient already given a biological sample for research?

### Test version

After determination of the data set and a coding system, the Medical Informatics Group Frankfurt installed a test version of the registry for undiagnosed patients. This test version was evaluated in a preliminary manner in terms of content and ease of use. Multiple users entered data of randomly selected anonymized health records of the students’ clinic for patients without a diagnosis of the FRZSE repeatedly. Technical problems as well as issues concerning the content were listed and discussed. The data set and user interface were optimized according to the identified problems. As a result, a tested and proven to work version of the registry for undiagnosed patients has been set up for further testing and evaluation in clinical routine.

## Discussion

To ensure comparability of ‘undiagnosed patients’ registry entries, findings and symptoms have to be encoded. We evaluated three medical coding systems (SNOMED CT, HPO and LOINC) for their usefulness and feasibility.

For our purposes, finding exact matches for symptoms described by patients and clinical findings as mentioned in the health record is necessary.

With exact matches of 98% of tested medical terms, a mean number of five deposited synonyms, SNOMED CT seemed to fit our needs best.

HPO and LOINC provided 73% and 31% of exact matches of clinical terms respectively. Bringing in more generic terms for a defined symptom, with SNOMED CT 99%, with HPO 89% and with LOINC 39% of terms could be coded. One has to consider, though, that by using more generic or too specific terms the precise meaning of a symptom can be lost. For example: Raynaud’s phenomenon could be coded exactly with the SNOMED CT code “SCTID 266261006”. HPO provides only the more general codes for “cyanosis” (*HPO-Code* 0000961) or “abnormality of blood circulation” (*HPO-Code* 0011028) whereas the LOINC-code 67732-8 encodes only a very specific clinical situation, namely white finger syndrome or Raynaud’s syndrome caused by excessive vibration from pneumatic hammers or drills.

With a hit ratio of 31%, LOINC was not suitable for sufficiently coding symptoms and clinical findings in our test-cases.

One could argue that the evaluation of only 10 patient files for the comparison of the three coding systems, is not enough. The focus of our study was, however, to estimate the usefulness and feasibility of each nomenclature for the purpose of the registry. So, even when working with only a few cases, the strengths and limitations of each nomenclature according to the needs of the registry, appear quite clear.

Both the extraction of medical terms, their translation into English as well as deciding whether the term could be matched exactly or not with one of the coding systems was made by only one person. This makes our evaluation of the three coding systems potentially subject to errors. It would have been better, to have two researchers extract the terms and define the exactness of a match independently of each other. Possibly involving a third person who decides, when the results of the two researchers vary. We don’t assume, though, that many errors were made extracting the medical terms as working out the guiding symptoms is a daily task for clinicians. Deciding, whether a match is an exact one, does not seem difficult as it either is an exact match or not. Therefore, we think, that having assigned more researchers to these tasks would not have changed the results of our evaluation significantly.

The HPO ontology is extended continuously [43]. We expect the fraction of adequate hits of HPO to improve significantly over time. HPO is widely used for deep phenotyping in the field of rare diseases. The phenotype profile can be compared with computational disease profiles in the HPO database with the aim of identifying genetic diseases with comparable phenotypic profiles. Also, HPO provides for interoperability with other ontologies and it plays a key role with the Exomizer tool, which identifies potential disease-causing variants from whole-exome or whole-genome sequencing data [43, 44].

Taking into account HPO’s acceptable match rate of medical terms, the fact that it is available free of charge and especially its wide application and interoperability in the field of rare diseases, we think that HPO is the ontology of choice for an undiagnosed patients’ registry.

The European Common Data Set for Rare Disease Registration also recommends the phenotype of patients to be recorded with HPO [42]. In cases where a symptom cannot be coded adequately with HPO, a request can be sent to the developers of HPO to ask for the definition and addition of a new more suitable code to the HPO ontology for future use.

Although SNOMED CT proved to be the best fit for our needs, one limitation is the requirement of a national license, which is available in Germany only since the beginning of 2020 and for now only in the context of the Medical Informatics Initiative in Germany [45]. Furthermore, the current national license and use of SNOMED CT is still undergoing evaluation. However, as the German policy clearly strives for a permanent adoption, we take SNOMED CT into account in the context of future operations and further development of our registry.

Since OSSE as a registry toolkit allows for an uncomplicated modification of the registry’s forms and data elements, one of the first adjustments should be the inclusion of date of first contact with a specialized centre and a genetic diagnosis, coded by the international classification of mutations (HGVS) as suggested by the European Common Data Set for Rare disease registries. The inclusion of the patient’s disability profile according to the international classification of functioning and disability does not seem practical to us, as it is far too comprehensive to be implemented into the routine service of centres for rare diseases [42].

As OSSE is an open-source software, further developments and adjustments could be performed to meet specific needs of the distinctive nature of a registry for undiagnosed patients. Such changes could for example include statistics that would be calculated dynamically as the data base grows to show progress in the process of diagnosing patients or other key values.

Recurring issues in the field of registries in general and especially in the field of rare diseases are sustainability and ethical as well as legal concerns, particularly data protection regulations.

After initial funding of the software development by the European Commission, users of the OSSE software have to manage sustainable funding of such registries on a national level. In some European countries such funding is available within the framework of the respective national plan for rare diseases. In Germany, another means of funding can be through additional surcharges for particular tasks of specialized care centres. A resolution of the Gemeinsamer Bundesausschuss (G-BA) as of November 20th, 2020 has defined the implementation and/or conduction and evaluation of a registry for rare diseases as one of several specialized tasks of centres for rare diseases [46, 47].

Another critical point is the establishment of our proposed registry in different centres for rare diseases across Germany and possibly Europe. Every research site has to examine itself, whether a project like this registry meets all the ethical and legal requirements. A crucial point is data ownership and data sharing. A request to share identifiable patients’ data, even in the framework of a joint research project, leads to inquiries at the legal department and the data protection office in most cases. Therefore, we favour a decentral approach of multiple registries at different sites using the same metadata and data sets, that enables joint data evaluation using only de-identified data. The data collected by each registry remains in the custodianship of each site. Another consideration is the ongoing activity to establish registries for undiagnosed patients in several rare disease centres. Most likely, such separate registries may have their own primary objective. As the parallel existence of multiple registries usually leads to expensive efforts for the necessary data integration, we think it is crucial, that such registries are respecting existing standards of data schemes and support data integration. Our proposed registry, set up based with the OSSE toolbox, could serve as a blueprint and joint minimal data set for such registries. Each site is free to enlarge its own registry application with additional data elements, e.g., such as indicators regarding patient journeys, patient satisfaction or quality of life. It is important to involve patient organizations, who are one of the key stakeholders, in developing these to make sure, that the data elements are meaningful from their position, too. Enabling other centres to use these extensions can increase the data base for collaborative data evaluation. Therefore, it would be very helpful, if every registry site shared their metadata in publicly accessible repositories, which can easily be accomplished with the OSSE metadata repository. Furthermore, to be found by the community or whoever is interested, every registry should be enlisted in a registry of registries, i.e. the European Rare Disease Registry Infrastructure Directory of Registries [48].

## Conclusions

With the use of the OSSE software and a data set which focuses on symptoms and clinical findings, a functioning and meaningful registry for undiagnosed patients can be implemented. The next step is the utilization of the registry in centres for rare diseases. The FRZSE is currently creating a retrospective registry containing the data of all its previous patients. This project will show, among other things, if the design of the registry suggested by us meets the needs in clinical routine and can be applied to a large number of patients. After evaluation and possible adjustments, we also plan to implement a multi-centre decentral prospective registry.

## Data Availability

The datasets used during the current study are available from the corresponding author on reasonable request.
